# Properties of bilateral spinocerebellar activation of cerebellar cortical neurons

**DOI:** 10.3389/fncir.2014.00128

**Published:** 2014-10-27

**Authors:** Pontus Geborek, Fredrik Bengtsson, Henrik Jörntell

**Affiliations:** Neural Basis of Sensorimotor Control, Department of Experimental Medical Science, Lund UniversityLund, Sweden

**Keywords:** granule cells, spinocerebellar tracts, Golgi cells, Purkinje cell, mossy fiber, sensorimotor control, *in vivo* whole cell recording

## Abstract

We aimed to explore the cerebellar cortical inputs from two spinocerebellar pathways, the spinal border cell-component of the ventral spinocerebellar tract (SBC-VSCT) and the dorsal spinocerebellar tract (DSCT), respectively, in the sublobule C1 of the cerebellar posterior lobe. The two pathways were activated by electrical stimulation of the contralateral lateral funiculus (coLF) and the ipsilateral LF (iLF) at lower thoracic levels. Most granule cells in sublobule C1 did not respond at all but part of the granule cell population displayed high-intensity responses to either coLF or iLF stimulation. As a rule, Golgi cells and Purkinje cell simple spikes responded to input from both LFs, although Golgi cells could be more selective. In addition, a small population of granule cells responded to input from both the coLF and the iLF. However, in these cases, similarities in the temporal topography and magnitude of the responses suggested that the same axons were stimulated from the two LFs, i.e., that the axons of individual spinocerebellar neurons could be present in both funiculi. This was also confirmed for a population of spinal neurons located within known locations of SBC-VSCT neurons and dorsal horn (dh) DSCT neurons. We conclude that bilateral spinocerebellar responses can occur in cerebellar granule cells, but the VSCT and DSCT systems that provide the input can also be organized bilaterally. The implications for the traditional functional separation of VSCT and DSCT systems and the issue whether granule cells primarily integrate functionally similar information or not are discussed.

## Introduction

Dorsal and ventral spinocerebellar tract (DSCT and VSCT) cells are traditionally viewed as components of two separate systems. Ventral spinocerebellar tract projections ascend in the contralateral lateral funiculus (coLF) of the spinal cord whereas the DSCT projections ascend in the ipsilateral LF (iLF). Dorsal spinocerebellar tract neurons were initially believed to originate primarily from Clarke’s column in the dorsomedial part of the spinal gray matter whereas VSCT neurons originate from neurons located more centrally in the spinal gray matter (Matsushita et al., 1979; Matsushita and Ikeda, 1980). However, the DSCT also originates from a large population of neurons located outside Clarke’s column and these neurons can be located anatomically not very far from VSCT neurons (Aoyama et al., 1988; Edgley and Gallimore, 1988; Edgley and Jankowska, 1988; Shrestha et al., 2012a,b). As spinocerebellar tract cells and spino-reticulocerebellar neurons presumably sample components of sensorimotor functions that are expected to be distributed in the spinal circuitry (Spanne and Jörntell, 2013) a minor anatomical separation could imply that these DSCT and VSCT neurons sample information from functionally related pools of spinal interneurons, which would suggest a less distinct functional subdivision of the two systems.

Granule cells of the cerebellar cortex have been suggested to primarily or exclusively sample mossy fiber information from functionally similar input sources individually (Jörntell and Ekerot, 2006; Bengtsson and Jörntell, 2009), whereas functionally varied information, for example representing different spinal sensorimotor functions, would consequently instead be distributed within the population of local granule cells (Spanne and Jörntell, 2013).

The sublobule C1 of the cerebellar posterior lobe is functionally interesting since its input from the VSCT is exclusively from the Spinal border cell (SBC) component of that tract (Matsushita and Ikeda, 1980). In addition, sublobule C1 seems to receive DSCT input exclusively from the dorsal horn component (dh) of the DSCT (Matsushita and Ikeda, 1980). As judged by the peripheral input to the climbing fibers, the sublobule C1 is located in between lobules that process input from the forelimb and the hindlimb, respectively (Geborek et al., 2013b). To the extent that that the climbing fiber input reflects the motor functions of the cerebellar region, which is the case for the corresponding region in the cerebellar anterior lobe (Gibson et al., 1987; Robinson et al., 1987; Ekerot et al., 1995; Jörntell and Ekerot, 1999), the sublobule C1 would hence be expected to be primarily concerned with the control of proximal limbs and the torso.

In the present study, we activated the DSCT and the VSCT by stimulating the LFs of the two sides separately and recorded the responses of the cerebellar neurons in the left sublobulus C1 of the cerebellar cortex. Whereas some of the granule cells responded intensely to input solely from one of the sides, others responded to input from both sides. However, in the latter cases the temporal topography of the early phase of the responses evoked from the two sides were typically similar and simultaneous activation of the two sides did not result in summated responses. This was highly surprising and prompted us to also explore the possible cells of origin of these two tracts in the lumbar spinal cord. Whereas putative SBC neurons and putative dh DSCT neurons responded antidromically to coLF and iLF stimulation, respectively, a substantial part of the cells in the same region was found to have dual projections through the LFs. The existence of bilaterally projecting spinocerebellar neurons could explain the bilateral LF responses in granule cells. Bilaterally organized inputs could make sense functionally for sensorimotor information that is related to the control of the torso, an inherently bilateral task. In addition, our findings suggest that the functional differences between the VSCT and the DSCT systems could be less distinct than generally believed.

## Materials and methods

The experimental procedures were approved in advance by the local Swedish Animal Research Ethics Committee. The preparation and the recordings were the same as those previously described (Geborek et al., 2013b) and the reader is referred to that publication for details. Initial surgery was performed under propofol anesthesia, and all efforts were made to minimize suffering. Our EEG recordings were characterized by a background of periodic 1–4 Hz oscillatory activity, periodically interrupted by large-amplitude 7–14 Hz spindle oscillations lasting for 0.5 s or more. These forms of EEG activities are normally associated with deep stages of sleep (Niedermayer and Lopes Da Silva, 1993). The pattern of EEG activity and the blood pressure remained stable and did not change with noxious stimulation throughout experiments. The present material comprised *N* = 12 adult cats. An important difference with our previous report was that we compared the responses evoked from the contralateral funiculus to those evoked from the ipsilateral funiculus, which represent the VSCT and the DSCT, respectively. Therefore, at the level of the thoracic spinal cord segments Th7-8 we placed one stimulation microelectrode inside the coLF and another one in the iLF. Cerebellar cortical neurons of the C1 zone of the sublobule C1 were recorded from using patch pipettes with a potassium gluconate-based internal solution. Five of our granule cells and three of our Golgi cells were recorded in the intracellular whole cell current clamp mode, the rest of the cerebellar neurons were recorded in the loose-patch cell-attached mode for extracellular spike recordings. Granule cells, Golgi cells and Purkinje cells (PCs) were identified as previously described (Geborek et al., 2013b). Briefly, the identification of a unit as a granule cell was primarily based on the presence of interspike intervals of <2.0 ms, and by verifying that they were located in the granule layer based on field potential recordings (Bengtsson and Jörntell, 2007) and by keeping track of the depths at which PCs were encountered in each experiment for each plane of penetration (this could be done since each experiment involved a high number of electrode tracks). Golgi cells were identified by their long tuning distances and the absence of interspike intervals <2.0 ms. Purkinje cells were identified by their location in the Purkinje cell layer (as judged from field potentials) and the presence of complex spikes. All these recordings were made in the whole cell mode or in the loose patch cell-attached mode. Spinal neurons of the lumbar spinal segment L4 were recorded extracellularly using custom-made tungsten-in-glass microelectrodes (10–30 µm of exposed tungsten at the tip).

### Analysis

As in our previous paper, we used both single pulse and triple pulse stimulation (0.1 ms pulse width, 3 ms interpulse interval, repeated at 1–3 Hz). The LFs were stimulated at 0.3 mA for quantification of responses. In order to verify absence of input recorded at 0.3 mA, we sometimes used intensities of up to 1 mA. We included only granule cell responses that occurred within 11 ms of the onset of the stimulation, counted from the first effective stimulation pulse, to avoid including potential contributions from other pathways than the spinocerebellar pathways (Geborek et al., 2013b). All responses and response latency times were quantified from peristimulus histograms. A response started at the first time bin when there was a deviation of more than one standard deviation compared to the 100 ms prestimulus baseline activity for at least three out of five consecutive bins. An exception to this rule was a few cases of granule cells with substantial responses (>> 2 standard deviations of prestimulus baseline) for less than three bins.

## Results

### Granule cell recordings

We explored the distribution of responses evoked from the iLF and coLF in neurons of the cerebellar cortex *in vivo*. As in a recent study, all cerebellar recordings were made in the C1 zone of the sublobule C1 of the posterior lobe (Geborek et al., 2013b), where the VSCT input is represented selectively by the SBC component of the VSCT and where DSCT input is also present (Matsushita and Ikeda, 1980). We start our account with the recordings from the granule cells.

**Figure 1:**
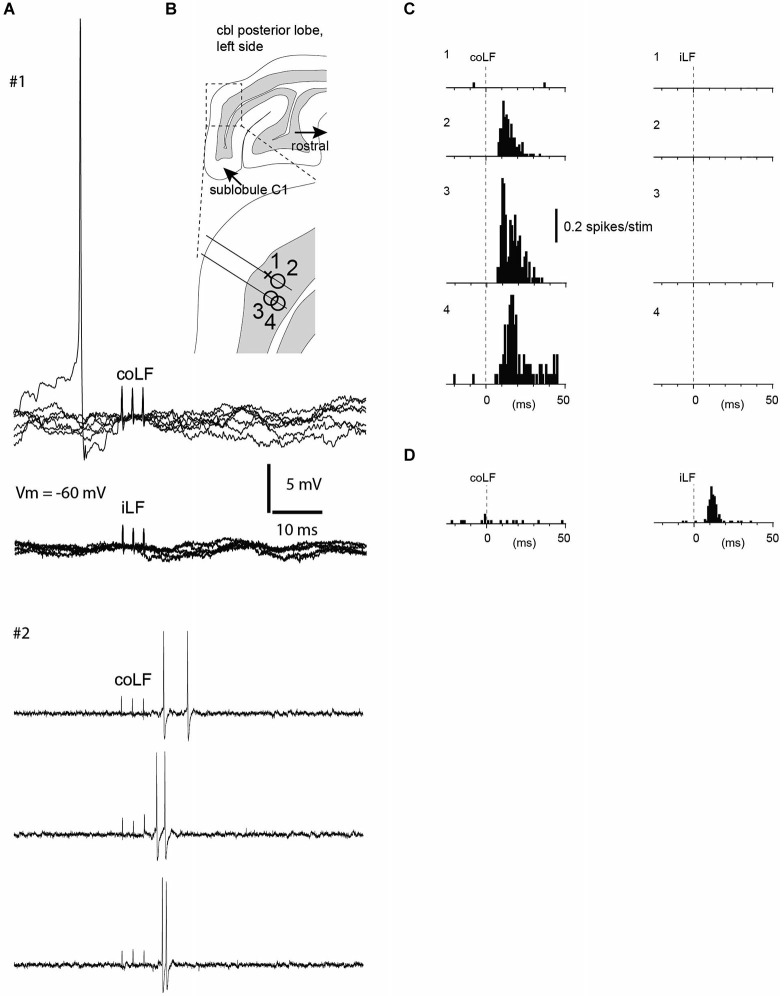
**Granule cells without responses to coLF and iLF were located nearby granule cells with strong responses**. **(A)** Sample traces from two neurons recorded in the same track (neurons #1 and #2, see panel **B**). Neuron #1 was a whole cell recording from a granule cell with no response to stimulation of either LF, as shown in the superimposed raw traces. Notice the absence also of a detectable subthreshold response in this case. Sample traces from neuron #2, a granule cell recorded in the cell-attached mode, which responded to the coLF stimulation only, are shown below. **(B)** Location of four granule cells recorded in the vicinity of each other within sublobule C1. The cross indicates the non-responsive granule cell illustrated in **(A)**, the circles indicate three other granule cells that did respond to LF stimulation. **(C)** Peristimulus histograms of the spike responses of the four granule cells in **(B)**. Granule cell #1 had no response, whereas the other three granule cells, in which we could not gain intracellular access, had strong responses to coLF stimulation. None of the cells responded to iLF stimulation. **(D)** A granule cell from another recording site that responded only to iLF stimulation.

**Figure 2:**
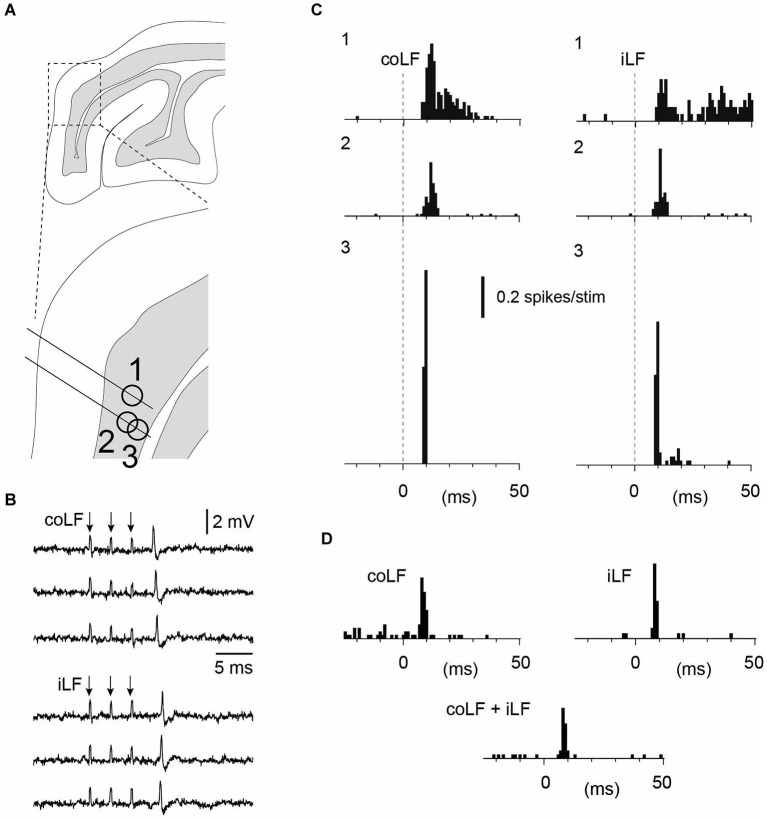
**Granule cells with responses to both iLF and coLF stimulation**. **(A)** Approximate recording location of the three granule cells in sublobule C1. Same experiment as in Figure 4. **(B)** Sample raw traces of neuron #3 to coLF and iLF stimulations, respectively. **(C)** Peristimulus histograms of the spike responses of granule cells #1–#3 to coLF and iLF stimulation, respectively. **(D)** Peristimulus histograms of a fourth granule cell in the same experiment with input from both coLF and iLF. In this case the LFs were stimulated with a single pulse each. In the middle histogram, both the coLF and the iLF were stimulated simultaneously. Note the absence of summation, indicating that the coLF and iLF stimulations activated the same mossy fibers.

As in our previous study, the majority of granule cells in this region did not have any response at all to stimulation of the LF, regardless of which side was stimulated (only 27 out of 129 granule cells responded to stimulation of either LF in this investigation). For five of the non-responding granule cells we obtained intracellular recordings in the whole cell mode—in each of these cases, responses were completely absent, i.e., there was neither excitatory nor inhibitory responses in the membrane potential within the first 20 ms after the onset of the stimulation to either of the LFs (iLF and coLF, respectively; Figure 1A). Strikingly, within the same granule layer, 10–150 µms away from such non-responsive granule cells, we found granule cells (and Golgi cells, cf. below) with prominent responses to LF stimulation (Figure 1B). In addition, granule cells with strong responses to coLF stimulation (Figure 1C) could be found in the same track as granule cells with strong responses to both coLF and iLF stimulation (Figures 1D, 2). Out of the 25 granule cells with responses to LF stimulation, 37% (10/27) responded to both iLF and coLF, 41% (11/27) responded only to coLF and 22% (6/27) responded only to iLF stimulation. As a rule, the cells responded with high-intensity, although exceptions were found.

**Figure 3:**
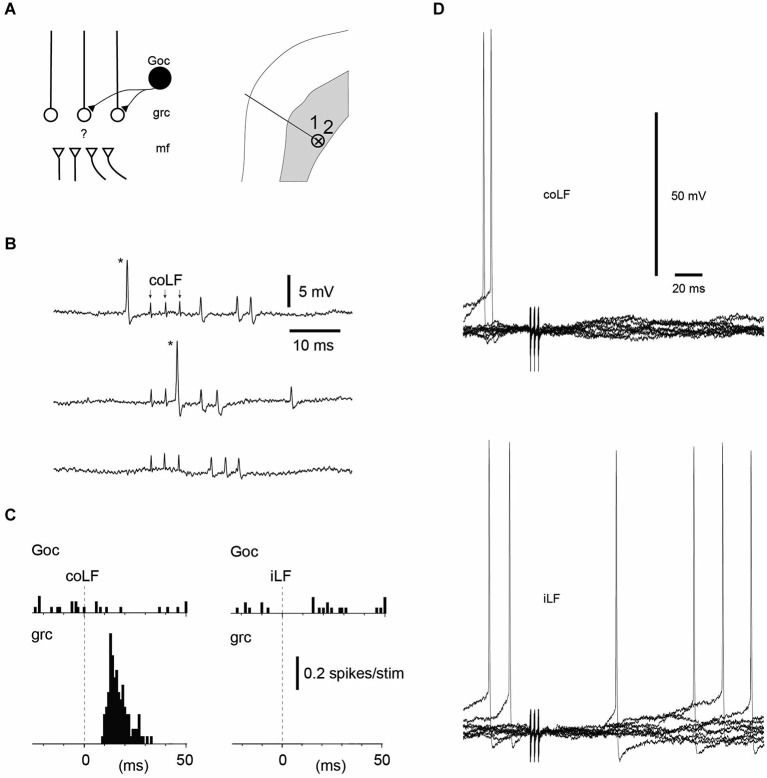
**Golgi cells without responses to coLF and iLF stimulation could be located nearby granule cells with responses**. **(A)** Recording location in the sublobule C1 of a Golgi cell and a granule cell recorded simultaneously, in the extracellular cell-attached mode. **(B)** Sample responses of the Golgi cell and the granule cell to coLF stimulation (3 pulses, 333 Hz; shock artefacts, indicated by arrows in the top trace. Asterisk, the spike of the Golgi cell. **(C)** Peristimulus histograms of the spike responses of the Golgi cell and the granule cell to stimulation of the coLF and the iLF, respectively. **(D)** Whole cell Golgi cell recording from another Golgi cell without responses to the iLF and the coLF. Similar to the intracellular granule cell recording (Figure 1A), note the absence also of subthreshold membrane potential changes.

**Figure 4:**
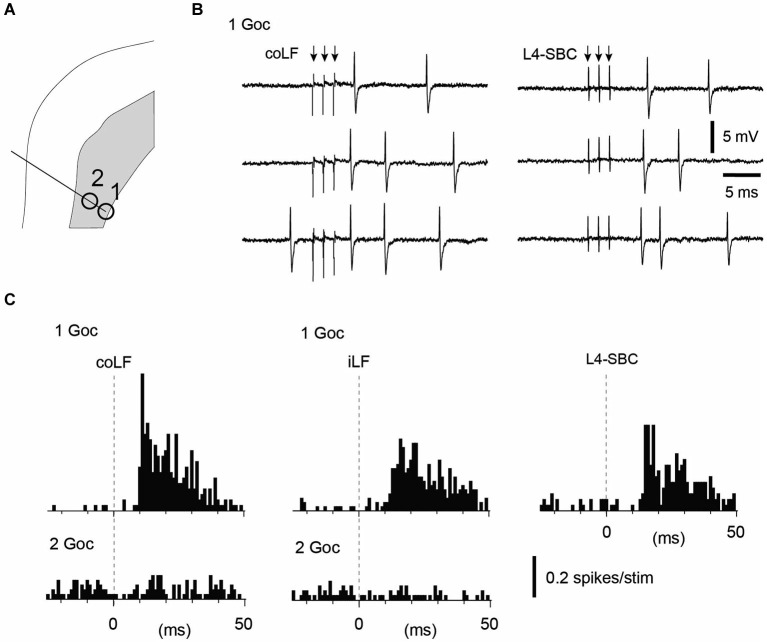
**Golgi cells recorded in the same track could vary with respect to their input from the LFs**. **(A)** Recording location in the sublobule C1 of two Golgi cells in the same electrode track. **(B)** Sample raw traces of evoked responses in Golgi cell #1. **(C)** Peristimulus histograms of the spike responses of the two Golgi cells to stimulation of the coLF and the iLF, respectively. One of the Golgi cells displayed a strong response to both LFs, the other one did not respond at all. For the Golgi cell with responses only, we tested also input from the L4 segment of the spinal cord (same location as in Figure 1, where putative SBCs were recorded). The response evoked from the L4 segment had a similar topography and amplitude as the responses evoked from the LFs.

Interestingly, for granule cells responding to both iLF and coLF, the temporal response profile and response magnitude to the two inputs were strikingly similar, even though the responses differed between adjacent granule neurons (Figure 2C). A possible summation effect of simultaneously stimulated coLF and iLF inputs were tested for 3/10 of our granule neurons. Importantly, in none of these three cases could we observe any apparent summation of the two inputs, suggesting that the coLF and iLF stimulations activated the same mossy fiber axons at the level of the spinal cord (Figure 2D). In addition, the latency times of the responses evoked by the two stimuli differed by about 2 ms (absolute response latency time differences: 1.8+/−1.3 ms, *N* = 10), which suggests that if the LF stimulations were activating different branches of the same axons, the branch point of those axons were, on average, located closer to the Th7-8 location of the iLF and coLF stimulation electrodes than the L4 segment. This would suggest that at least the earliest bilateral responses were mediated in part by neurons located in the lowest thoracic segments or upper lumbar segments. But as the responses evaluated were the granule cell spike responses to a brief burst stimulation of the LFs, this measure is naturally not a strong indicator of the location of the spinocerebellar neurons.

### Golgi cell recordings

We next looked at the Golgi cells (*N* = 18 cells tested for input from both funiculi). Similar to the granule cells, the majority of the Golgi cells (11/18) lacked spike responses to stimulation of either of the LFs, even though they in some cases were located just next to a granule cell or another Golgi cell that did respond (Figures 3A–C). Out of the non-responding Golgi cells, three were recorded in the intracellular whole cell mode. Again, similarly to the granule cells, non-responding Golgi cells did not display any subthreshold membrane responses either (Figure 3D). Golgi cells that did respond represented a mixed set (Figure 4)—half of them responded to both iLF and coLF (3/7), whereas the other half was predominantly responding to the coLF (3/7) rather than the iLF (1/7). For three of the Golgi cells that did respond to coLF (alone and/or also receiving input from the iLF), we tested input evoked by local microstimulation in the SBC region of the L4 segment. In all cases tested, the response to L4-SBC stimulation had similar temporal profiles as the responses evoked by the LFs, although there was a delay of 1–2 ms relative to the former.

### Purkinje cell recordings

**Figure 5:**
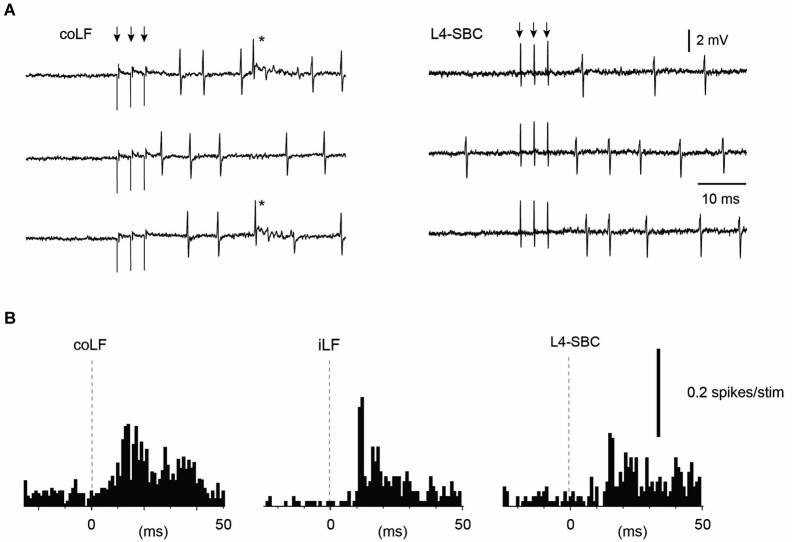
**Sample Purkinje cell responses to stimulation of the LFs and stimulation of the L4 segment**. **(A)** Sample raw traces. Asterisk indicates complex spike. **(B)** Peristimulus histograms. The Purkinje cell responded to both of the LFs, and the response to the L4 segment stimulation had a similar topography and amplitude as the LF stimulations. Only simple spike responses were analyzed.

Our final set of recordings was obtained from PCs. Since PCs are expected to pick up information from a large number of granule cells, they would be expected to respond to both funiculi as a rule. And this was indeed also found to be the case—9 out of 11 PCs responded to both the iLF and coLF, whereas the remaining two PCs were found to not respond at all (Figure 5). For three of these PCs, input form the L4-SBC was tested and, similar to the Golgi cells above, in each of these cases the L4-SBC stimulation evoked responses with a similar temporal profile and magnitude as the LF stimulations (Figure 5).

### Spinal cord recordings

**Figure 6:**
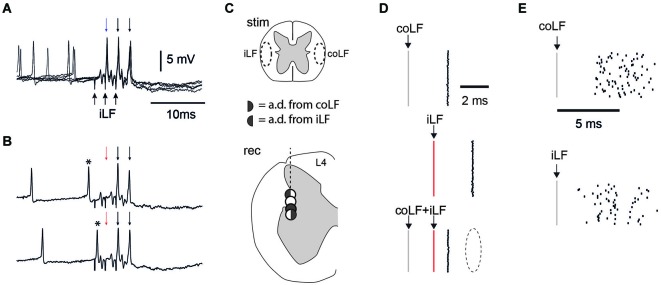
**Recordings in the L4 segment of the spinal cord**. **(A)** Spinal neuron with an antidromic response to iLF stimulation. Each stimulation pulse invariably elicited a spike. In this case, the recording electrode could elicit spontaneous discharge in the otherwise silent neuron, as it was pushed against the cell to obtain a partially intracellular recording. Still, in these five superimposed traces, there was no spike jitter in the antidromic activation (arrows at the top) to three pulses (at 333 Hz) of iLF stimulation (arrows at the bottom). **(B)** The effect of spontaneous discharge within the time window of two times the antidromic response latency time (asterisk) was that it invariably blocked (one of) the antidromic spikes (red arrow). **(C)** Patterns of antidromic activation in four spinal neurons recorded in sequence in the same electrode track, in the vicinity of the lateral border between the spinal gray matter and white matter. One of the neurons (white circle) responded only synaptically to stimulation of either of the LFs (Cf. panel **E**), whereas the other three neurons responded to either LFs or both as indicated by the key in the middle of the panel. The three neurons were recorded from within 0.2 µm distance as measured from the depth reading of the step motor. **(D)** Collision test confirms dual projection neurons in the L4 segment. The two topmost raster plots illustrate the spike latency times for antidromic activation from the coLF and the iLF, respectively. Note the lack of spike jitter. The raster plot at the bottom illustrates that stimulation of both the coLF and the iLF, with the iLF stimulation being delayed relative to the former, invariably resulted in a block of the response to the iLF stimulation (dashed ellipse). **(E)** For comparison, the spike jitter of a neuron that was synaptically activated by the coLF and iLF stimulations in the same region of the L4 segment and in the same experiment.

Given the results above, which suggested a bilateral distribution of some of the spinocerebellar axons in the LFs, we next explored whether neurons located nearby SBCs in the L4 segment could have ipsilateral projections through the LFs. Stimulation of the coLF antidromically activate neurons located in the SBC region (Shrestha et al., 2012a) of the L4 segment (Geborek et al., 2013b) as well as VSCT neurons (Geborek et al., 2013a) and the same neurons can be antidromically activated from the cerebellum (Geborek et al., 2013b). As neurons of the DSCT have axons that ascend in the dorsal part of the iLF, we here tested the neurons recorded in the lateral part of the L4 segment, at depths between 1.50–2.00 mm from the dorsal surface of the spinal cord, for antidromic activation from the LFs of both sides (Figure 6). As previously reported, these projection neurons had no spontaneous firing frequency so the identification relied in most cases on the absence of spike jitter to repeated high-frequency stimulation trains of the fiber tract (Geborek et al., 2013b; Figure 6A). In some cases, pushing the recording electrode against the recorded neuron a few µms could elicit spontaneous spikes. When these spontaneous spikes preceded the antidromic invasion evoked through LF stimulation, within the time window defined by the antidromic response latency time, the spike was always collided out (Figure 6B, observed in *N* = 5 neurons). This investigation provided the surprising result that a fair number of the neurons that were antidromically activated and located nearby the SBC region of the L4 segment was found to project through BOTH the iLF and the coLF (Figure 6C). Among the neurons recorded (*N* = 32) about one third projected through iLF (11/32, 34%, recorded at depths of 1.50–1.65 mm), one third through the coLF (9/32, 28%, recorded at depths of 1.65–2.00 mm), and one third through both the iLF and coLF (12/32, 38%, recorded at depths of 1.60–1.80 mm). In the case of double projecting cells, we could in addition verify their antidromic activation from both the iLF and the coLF by collision tests (this was done in *N* = 2 cases; Figure 6D). The response latency times for antidromic activation was 2.17+/−0.70 ms for coLF (*N* = 23) and 2.14+/−0.82 ms for iLF (*N* = 22) (data omitting four outliers, with antidromic latency times greater than 5 ms). For bilateral projection neurons, the differences in antidromic latency times from the two LFs was less than 0.2 ms. In contrast to antidromically activated cells, in local spinal neurons that were found to be only synaptically activated from the iLF and/or the coLF (*N* = 22 neurons), spike jitter (defined as a standard deviation of >> 0.1 ms) was always observed in the evoked response (Figure 6E).

## Discussion

**Figure 7:**
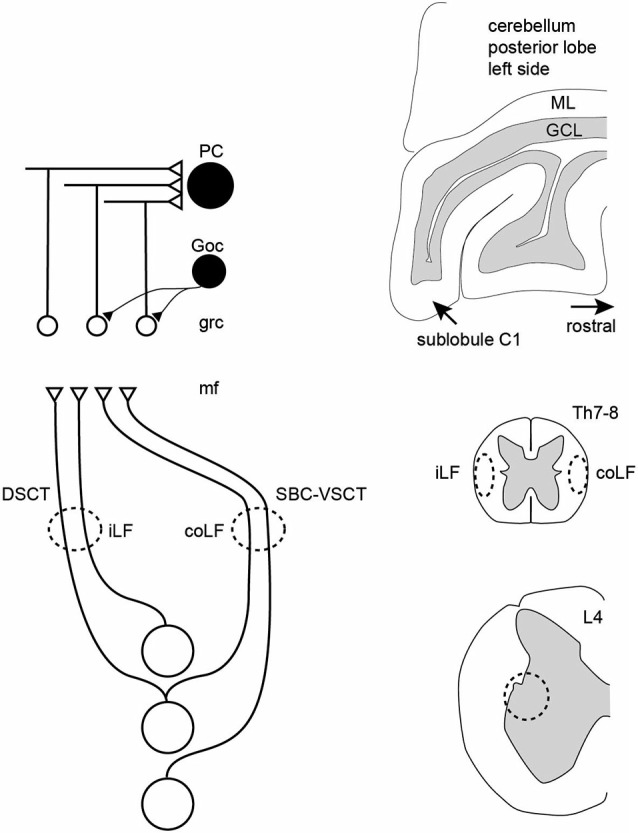
**Schematic summary of the results**. In the paravermis of the cerebellar sublobule C1, granule cells can respond to either the iLF, per definition the dorsal spinocerebellar tract (DSCT) or the coLF, per definition the ventral spinocerebellar tract. In addition, some granule cells responded to both LFs but then both the LFs produced a response with similar temporal topography and amplitude, and the responses from the two LFs did not summate. A conclusion was therefore that a spinocerebellar neuron can project through both funiculi, and this was also confirmed in direct recordings from spinal neurons located within a known point of origin of spinocerebellar neurons. Both PCs and Golgi cells responded preferentially to inputs from both of the LFs, consistent with their larger number of excitatory inputs. PC, Purkinje cell; Goc, Golgi cell; grc, granule cell; mf, mossy fiber; ML, molecular layer; GCL, granule cell layer; Th7-8, thoracic segment 7–8 of the spinal cord; L4, lumbar segment 4 of the spinal cord.

The present paper illustrates that granule cells of the sublobulus C1 of the cerebellar posterior lobe can receive input from either the coLF, the iLF or, for a subpopulation, both. However, in the latter case the responses that were evoked from the two sides were similar and did not summate, suggesting that the bilaterally evoked responses were at least partly generated by the same set of spinocerebellar axons. In addition, we found that spinal neurons located in the vicinity of the SBC region of the L4 lumbar segment can, besides the normal coLF projection typical for VSCT neurons, alternatively have a projection up through the iLF (as expected for dh DSCT neurons) or even have a projection that ascends through both the iLF and the coLF (Figure 7). These results have implications for the traditional separation of the DSCT and VSCT systems as two functionally separate pathways and provide a new development in the current debate on the function of granule cells as integrators of disparate or related information.

### Granule cell response patterns and the converging spinocerebellar neurons

As granule cells *in vivo* typically require co-activation of three or four mossy fiber synapses to trigger a spike (Jörntell and Ekerot, 2006), the temporal topography of the responses evoked through electrical tract stimulation should to a large degree be determined by the distribution of the response latency times of the particular mossy fibers that converge on a given granule cell (Spanne et al., 2014a). Using fiber tract stimulation, a difference in response topography between two adjacent granule cells (Figures 1, 2) could consequently depend on that the population of mossy fibers that converge on one of the cells have a similar response latency time whereas the mossy fibers converging on the other cell have a more distributed set of response latency times. At least at this short time scale, Golgi cell inhibition would not be expected to influence the temporal pattern of the granule cell responses (Jörntell and Ekerot, 2006; Bengtsson et al., 2013). Hence, the similar response topographies evoked from the two sides seemed to suggest that the same number of mossy fibers, with the same combination of conduction velocities, were evoked from both sides. This seemed to be an unlikely coincidence and in the three cases where we tested for summation of the iLF and coLF inputs they did not summate, suggesting that we in these cases activated the same population of axons from the two sides. This could be explained if some of the spinocerebellar projection neurons had bilaterally ascending axons, which could be activated from either LF. The existence of such spinocerebellar cells could also be confirmed in our experiments where we recorded from the L4 segment of the spinal cord. The relatively small response latency time differences of the granule cell responses evoked from the two sides provided additional information about the approximate location of the spinal neurons that provided the spinocerebellar input. As we stimulated in the Th7-Th8 segments, and the antidromic latency time to the L4 SBC was larger than the difference in response latency times in the granule cells, at least part of the responses we evoked should have been generated by spinocerebellar neurons in the thoracic segments (Matsushita et al., 1979; Matsushita and Ikeda, 1980; Yaginuma and Matsushita, 1987). Notably, as we stimulated the lower part of the thoracic cord, some of the many granule cells that did not respond at all could possibly have received spinocerebellar input from higher spinal cord levels. The Golgi cells and PCs recorded overall seemed to integrate granule cell inputs in a linear fashion (Figures 4, 5), in line with their linear encoding properties (Spanne et al., 2014b).

The existence of dual projection spinocerebellar cells has not been explicitly reported in the literature as far as we know, although putative examples are the “ambigious” spinocerebellar projection neurons of the central cervical nucleus (Hirai et al., 1984). This could in part be explained by that most studies of these systems over the last 50 years have implicitly assumed an unilateral projection and have only looked for neurons with projections through the relevant funiculus. Another possibility is that among our neurons with dual ascending LF projections, only one of the branches may be travelling all the way to the cerebellum, whereas the other branch could be involved in spinal integration of information, as recently demonstrated for VSCT neurons (Geborek et al., 2013a).

### Implications for the function of spinocerebellar neurons

The origin of DSCT from neurons located in Clarke’s column as opposed to the origin of VSCT from neurons located more ventrally and laterally in the spinal cord and early findings of a cutaneous input to the DSCT but not the VSCT has inspired a view that these constitute two separate ascending systems. However, both systems forward information on actions of premotor spinal interneurons from muscle afferents to alpha-motorneurons (Krutki et al., 2011). In relative terms, the number of excitatory terminals from motor command structures is higher in VSCT than in DSCT neurons (Shrestha et al., 2012a) but the influence of motor command signals on DSCT neurons is nevertheless strong (Hantman and Jessell, 2010; Fedirchuk et al., 2013). In addition, as the location in the spinal cord would be expected to reflect the type of spinal interneuron input the spinocerebellar neurons sample, the minor separation in anatomical location of some of the DSCT and VSCT neurons suggests that the functional differences between them could be less distinct than generally believed. Instead, the anatomically subdivided spinocerebellar tract systems may contain a continuum of spinal cord sensorimotor functions (Spanne and Jörntell, 2013) where some types of functions are preferentially, but not exclusively, distributed to a particular system, whereas other functions are common to all systems.

Why would spinocerebellar neurons have bilateral projections? A main explanation could be that these neurons are not only used as information channels informing the cerebellum about the activity in the spinal motor circuitry, but the same fibers could also be used for providing input to other spinal interneurons (Geborek et al., 2013a) and neurons of the brainstem and thalamus (Johansson and Silfvenius, 1977a,b; Huber et al., 1994). A bilateral projection would be useful if the same fiber is used for example by brainstem nuclei on either side of the midline, as would be expected from the spinocerebellar information destined for the cerebellar sublobulus C1, which appear to be related to proximal limb and torso motor control (Geborek et al., 2013b).

### Implications for the function of cerebellar granule cells

Early ideas about the function of the granule cells were that they would carry out expansion recoding and, consequently, that each granule cell should sample a unique combination of mossy fiber inputs, that the granule cells should represent as diverse combinations of inputs as possible and that the coincident activation of the four mossy fiber inputs was required to generate granule cell spike output (Marr, 1969). But the idea of expansion recoding in cerebellar granule cells were formulated without considering that such recoding could possibly take place already within the mossy fiber systems themselves. At least spinocerebellar systems are known to represent a wide spectrum of integrated sensorimotor signals (Spanne and Jörntell, 2013). These integrated sensorimotor signals are the result of processing in a relatively complex neuronal network in the spinal cord and have a close correspondence to the type of information that the cerebellum would need in order to achieve limb intersegment coordination, for example. Given the relatively highly processed nature of this information, and its close connection to the state of the movement apparatus in relation to motor commands, it would not obviously be an advantage to mix this information with input from functionally disparate sources. The present paper provides another piece of evidence that the cerebellar granule cell layer of the paravermal cerebellar cortex appears to be segregated into compartments where functionally similar information is processed by the granule cells and Golgi cells. In addition, the findings also put the spotlight on that functionally related information may be derived from systems that have historically been viewed as functionally disparate.

## Conflict of interest statement

The authors declare that the research was conducted in the absence of any commercial or financial relationships that could be construed as a potential conflict of interest.
